# University MOOC should be added with farmer interested sections and provide individualized service to adapt to farmer training

**DOI:** 10.1371/journal.pone.0288309

**Published:** 2023-11-02

**Authors:** Zhi-ping Zhang, Bing Hua, Jie-xia Liu, Hai-bo Dai, Min-min Miao

**Affiliations:** College of Horticulture and Landscape Architecture, Yangzhou University, Yangzhou, Jiangsu Province, People’s Republic of China; Hebei Agricultural University, CHINA

## Abstract

Vegetables represent an important agricultural industry in China. New farmers and new technologies for vegetable production have emerged in recent years, which makes farmer training very necessary. On the other hand, massive open online courses (MOOCs) are currently widely used in universities. The purpose of this study is to investigate the importance of different sections of a university MOOC focused on olericulture to farmers with different demographic characteristics and provide a basis to improve university MOOCs for farmer training. The survey results suggest that the age, education level, gender, farmer scale, facility type and profit of farmer learners are important factors determining evaluations of the importance of different MOOC sections, indicating that services customized to different farmer populations are necessary. Among different sections of MOOC “Olericulture”, farmers with younger age, higher education, larger farm, more advanced facility and more profit were more interesting in sections include cultural, social and theoretical knowledge, and less interesting in practical skill sections. Based on the survey, eight new sections including one marketing subsection (new agricultural supplies and market news), one social subsection (laws and regulations), two practical subsections (practice videos, photos and videos from other farms), and three comprehensive subsections (discussion of practical issues, mechanization, and smart olericulture) were added to the original MOOC, and the results indicate that this improvement is efficient in enhancing the importance evaluations and profits of all farmer learners, especially among those with high education levels.

## Introduction

Vegetables are rich in vitamins and dietary fiber and are essential foods in people’s daily lives. To meet the demands of growing populations, the cultivated area of vegetables has increased rapidly in recent years worldwide [1]. China is the largest producer and consumer of vegetables in the world, and China’s current vegetable production area spans more than 20 million hectares (ha) [2]. The yield and cultivated area of vegetables in China are estimated to represent 58.31% and 52.25% of the world’s total vegetable yield and area, respectively, according to the Food and Agricultural Organization of the United Nations (https://www.fao.org/faostat/zh/#data/QCL) [3]. In addition, to ensure the annual supply and improve the per-unit yield, greenhouse vegetable cultivation, which always requires more techniques than open field vegetable production, has expanded rapidly in recent decades. It is estimated that more than 4.0 million ha of plastic sheds are being used in China, accounting for approximately 80% of the global plastic-covered vegetable production area [4].

Although China’s vegetable industry has made marked achievements, new situations and problems are still emerging. First, the aging of the labor force has accelerated significantly in the last ten years in China. Both a decreased birth rate and worker migration toward cities have contributed to a severe shortage of the working-age labor in rural regions [5]. As a result, the mechanization and intellectualization of vegetable management have become increasingly important, requiring farmers to renew their knowledge of traditional vegetable production [6]. On the other hand, fierce competition and high living costs in cities have led to some migrant workers returning to their home villages [7]. Some returned migrant workers and entrepreneurs who have accumulated a certain amount of wealth intend to employ new vegetable species and technologies or expand their farm scale. However, their lack of knowledge may significantly limit the realization of their ideas. In addition, according to ecological civilization, carbon peaking and carbon neutrality goals and other national strategies, the Chinese government requires farmers to reduce chemical fertilizer and pesticide inputs, nitrogen leaching and greenhouse gas emissions, notably during vegetable production, which is also compelling farmers to adopt new equipment and technologies [8]. Taken together, it has become quite urgent to strengthen farmer training in rural regions of China.

In China, farmer training is principally organized by the agricultural extension stations of counties or districts. In recent years, to achieve the goals of the series of national strategies mentioned above, the government has increased financial allocation to support more farmer training programs. However, the lack of training teachers and suitable teaching resources have become the major factors limiting the farmer training project enhancement, especially in the vegetable production area [9]. Currently, professional university faculties in the agricultural sector represent major teacher resources for farmer training organized by extension stations. Off-line courses now serve as the main mode of farmer training [10]. In this occasion, college teachers need to frequently go to the countryside to teach, and farmers sometimes have to attend university courses when a training program is associated with multiple courses and faculties. However, the schedules of both college teachers and farmers are restricted by their works, i.e., college teaching and researching for agricultural faculties and farm work for farmers. On-line courses should be one of the ways to solve this problem [11]. In recent years, due to the COVID-19 pandemic, online training programs have become more popular [12]. However, the online learning resources for farm training are very scarce now in China.

The rapid development of information and communication technology has precipitated a series of distance learning patterns, among which massive open online courses (MOOCs) have attracted broad attention from educational institutions, especially in higher education [13, 14]. Unlike traditional synchronous, in-person courses, MOOCs provide students the opportunity to take part in learning more freely without being limited by space or time constraints and to access lectures from the world’s best teachers [15]. Therefore, the university MOOC may be a potential way to alleviate the shortage of teaching resources for farmer training. On the other hand, among MOOCs in different fields, MOOCs in agriculture seem less interesting for the publics and always attract fewer learners. On the Chinese University MOOC platform (https://www.icourse163.org/), MOOCs in agricultural areas include fewer than 150 courses, and the average number of registered students is always less than 1500. From this point of view, it is also necessary to improve agricultural MOOCs to attract more audiences.

Several reports have investigated the influence factors of MOOC extension. Meet et al (2022) studied the factors affecting MOOC adoption in India and found that price value, hedonic motivation, facilitating conditions, performance expectancy and effort expectancy had positive effects on the MOOC adoption [16]. Al-Adwan and his colleagues (2020, 2021) concluded that four types of competency, social, technical, self-mamagement of learning, and communication, were important elements determining MOOC acceptance of Jordanian students [17–19]. Fianu et al. found the qualities of teaching content and information provided by MOOC were the major factors in MOOC usage [20]. However, to date little researches have focused on the factors affecting the MOOC adoption of farmers.

It is not appropriate to introduce university MOOCs directly to farmers since the knowledge needs of farmers and university students are quite different. Several informal surveys of our group have shown that some vegetable farmers reveal less interests to the theoretical parts of the MOOC and require more practical technologies including in the MOOC. In this study, a MOOC focused on olericulture was chosen to investigate the interests of farmers of different ages, educational backgrounds, genders, farming scales, facility types and profits. Based on an informal survey, some sections which are considered potentially interesting to farmers were added. The original and the phase-improved MOOC of were introduced to the farmers, the farmers’ evaluations of the importance of sections of the two types of MOOCs were analyzed and the effect of MOOC improvement on the farmers’ profit was also evaluated. The aim of this study was to improve current university MOOCs to meet farmers’ training needs according to the survey results, and then provide more teaching resources with high quality for farmer training.

## Materials and methods

### Description of the study area

The studied MOOC was mainly promoted in Jiangsu Province, China, where the authors’ affiliated institution is located and where their extension activity was conducted. Jiangsu Province is located in eastern China (116°18′-121°57E, 30°45′-35°20′ N) and belongs to the East Asian monsoon climate zone with an annual rainfall level of 1000–1400 mm and an average temperature of 13.6–16.1°C (http://www.cma.gov.cn/). Jiangsu is China’s 4^th^ largest province of vegetable production, and the provincial vegetable production area in 2021 spanned over 145.3 million ha, approximately half of which is covered with plastic film. In addition, Jiangsu is one of the most developed provinces in China with a GDP per capita reaching 19.9 thousand dollars in 2021 (http://www.jiangsu.gov.cn/).

Jiangsu Province is divided into three sections, i.e., south Jiangsu, middle Jiangsu, and north Jiangsu, according to their natural and social conditions. The average temperatures for these three parts are: 15~16°C, 14~15°C, 13~14°C, respectively. To increase the representativeness of this study, the three counties or districts with relatively large vegetable-seeded area were selected in each section. In southern Jiangsu, these included Taicang (31°24′N, 121°10′E) and Zhangjiagang (32°87′N, 120°57′E) in Suzhou and Jintan (31°75′N, 119°57′E) in Changzhou. In central Jiangsu, the three counties included Rugao (32°40′N, 120°57′E) in Nantong, Jiangdu District (32°43′N, 119°55′E) and Yizheng District (32°27′N, 119°18′E) in Yangzhou. In northern Jiangsu, the three counties included Qingpu District (33°58′N, 119°03′E) in Huaian, Haizhou District (34°57′N, 119°12′E) in the city of Lianyungang and Yan du District (33°33′N, 120°13′E) in Yancheng.

### MOOC promotion, survey design and data collection

The MOOC focused on olericulture was provided online in 2019 on the Chinese University MOOC platform (https://www.icourse163.org/). The MOOC is divided into 4 sections and 11 subsections: a cultural section (cultivation history and cultural connotations), social section (the present situation and nutritive value), theoretical section (botanical characteristics, growth cycles, and favorable environments), and practical section (types and cultivars, facilities and seasons, cultivation techniques, and pest control). To test the possibility of applying this university MOOC to farmer training, we asked local extension stations to recruit vegetable farmers to complete the MOOC in the selected counties and districts mentioned above in 2020. During this period, we added eight subsections to the MOOC according to the suggestions of extension officers and farmer learners: one marketing subsection (new agricultural supplies and market news), one social subsection (laws and regulations), two practical subsections (practice videos, photos and videos from other farms), and three comprehensive subsections (discussion of practical issues, mechanization, and smart olericulture). To test the effect of this modification, both the original and modified MOOCs were introduced to other vegetable farmers in these areas in 2021.

At each sampling county or district, from the list of MOOC learners provided by the local governments, 40 to 50 vegetable farmers were randomly selected from both the original MOOC learner group and modified MOOC learner group. Finally, 400 farmers from each group were selected. A questionnaire was used to collect the respondents’ age, education, gender, farm scale facility type, and profit information and their evaluations of the importance of different sections of the MOOC. The details of the questionnaire are shown in S1 Table. To evaluate the effect of MOOC learning, the annual profits of the sampled farmers before and after MOOC training was also surveyed.

### Statistics

Correlations between different characteristics of farmer learners were calculated using the Pearson correlation coefficient at p ≤ 0.05. Education, gender and main facility type data were assigned values for calculation, as shown in S2 Table. Levels of importance ascribed to different MOOC subsections were divided into five levels: Very important, Important, Somewhat important, Not very important, and Not important at all, assigned scores of 100, 80, 60, 40, and 20, respectively. The importance ascribed to different MOOC subsections was calculated as the average of all answers from certain groups. The overall degrees of importance of the original and modified MOOCs were calculated as the average of all subsections evaluated by farmers. SPSS version 15.0 (SPSS, Inc., Chicago, IL, USA) was used for one-way ANOVA of all data. Differences between treated samples were evaluated at 0.01 probability levels using Duncan’s test.

### Ethics statement

Approval to investigate the farmers’ responses to questionnaire in S1 Table was granted by the rural revitalization of the modern agriculture steering committee of Jiangsu. We distributed the questionnaire to farmers in the nine regions of Jiangsu Province as mentioned above, and informed them that they have the right to refuse to participate in the survey verbally. Farmers who were willing to participate the survey filled in and submitted the questionnaire anonymously on an online survey software. Therefore, no one knows which farmers participate or did not participate in the survey and there is not any compulsion involved.

## Results

### Demographic information of the investigated vegetable farmers

The distributions of age, education, gender, farm scale, facility type and profit data of the selected vegetable farmers were calculated according to the data collected from the questionnaire. As shown in Table 1, the aging of the vegetable farmer population is pronounced in the selected counties and districts. Farmers older than 50 and 60 accounted for 72.5% and 30.4% of the total vegetable farmer population, respectively. In addition, relatively low education levels among these farmers were also found. The percentage of farmers with a high school education or lower was approximately 56.8%, while that of farmers with a graduate degree or higher was only 18.0%. It is worth mentioning that several farmers with a postgraduate degree were found in the population. Regarding gender, most of the investigated vegetable farmers were male (80.4%), indicating that manual labor may still be important for vegetable farm management. Farming scales was divided into 5 levels in this study, and we found that nearly 56.4% of farmers grew vegetables over less than 1 ha, indicating that small-scale farms were still the predominant form for vegetable production in Jiangsu Province. Walk-in tunnel (47.3%) and solar greenhouse (31.6%) facilities are two major facilities used for vegetable cultivation in Jiangsu Province, although a few production facilities with intelligent control devices are emerging. Finally, annual profits show that most of the selected vegetable farmers earned profits in the range of 10–20 thousand dollars per ha (53.8%).

**Table 1:** Demographic information of the investigated vegetable farmers.

Category	Subcategory	Number	
		Unmodified	Modified
Age	20–29	42	47
	30–39	59	57
	40–49	72	70
	50–59	106	104
	≥60	121	122
Education	High school or lower	223	231
	Undergraduate degree	103	99
	Graduate degree	65	63
	Postgraduate degree	9	7
Gender	Male	317	326
	Female	83	74
Farming scale (ha)	<0.5	129	122
	0.5–1	98	102
	1–3	75	77
	3–10	53	52
	>10	45	47
Main facility type	Open field	23	25
	Simple mulching	56	51
	Walk-in tunnel	187	191
	Solar greenhouse	126	127
	Intelligent facility	8	6
Profit (thousand dollars/ha)	<5	48	52
	5–10	69	66
	10–20	217	213
	>20	66	69

To better understand the effects of farmer demographic characteristics on evaluations of MOOC section importance, a Pearson’s correlation analysis was performed of the factors mentioned above (Table 2). Negative correlations existed between the age and education, and age and facility advance. Compared to male farmers, female farmers have higher education level, farmer scale, more advanced facilities and earn more profit. Farmers with higher education levels tent to have higher farming scale and more advanced facilities. Strong positive correlations were also observed between farming scale and facility advance, and facility advance and profit. In addition, no significant correlations were found between age and gender, age and farming scale, age and profits, education and profits, farming scale and profits, etc.

**Table 2:** Pearson’s correlation coefficient analysis of demographic information of the investigated vegetable farmers.

	Age		Education		Gender		Farming scale		Main facility type		Profit	
	Unmodified	Modified	Unmodified	Modified	Unmodified	Modified	Unmodified	Modified	Unmodified	Modified	Unmodified	Modified
Age	1	1										
Education	-0.82**	-0.85**	1	1								
Gender	0.18	0.27	0.67**	0.72**	1	1						
Farming scale	-0.42	-0.43	0.73**	0.74**	0.69**	0.72**	1	1				
Main facility type	-0.86**	-0.91**	0.82**	0.83**	0.78**	0.81**	0.76**	0.78**	1	1		
Profit	-0.43	-0.47	0.41	0.45	0.69**	0.63**	0.32	0.41	0.91**	0.88**	1	1

### Vegetable farmers’ evaluations of different olericulture MOOC subsections

**Fig 1:**
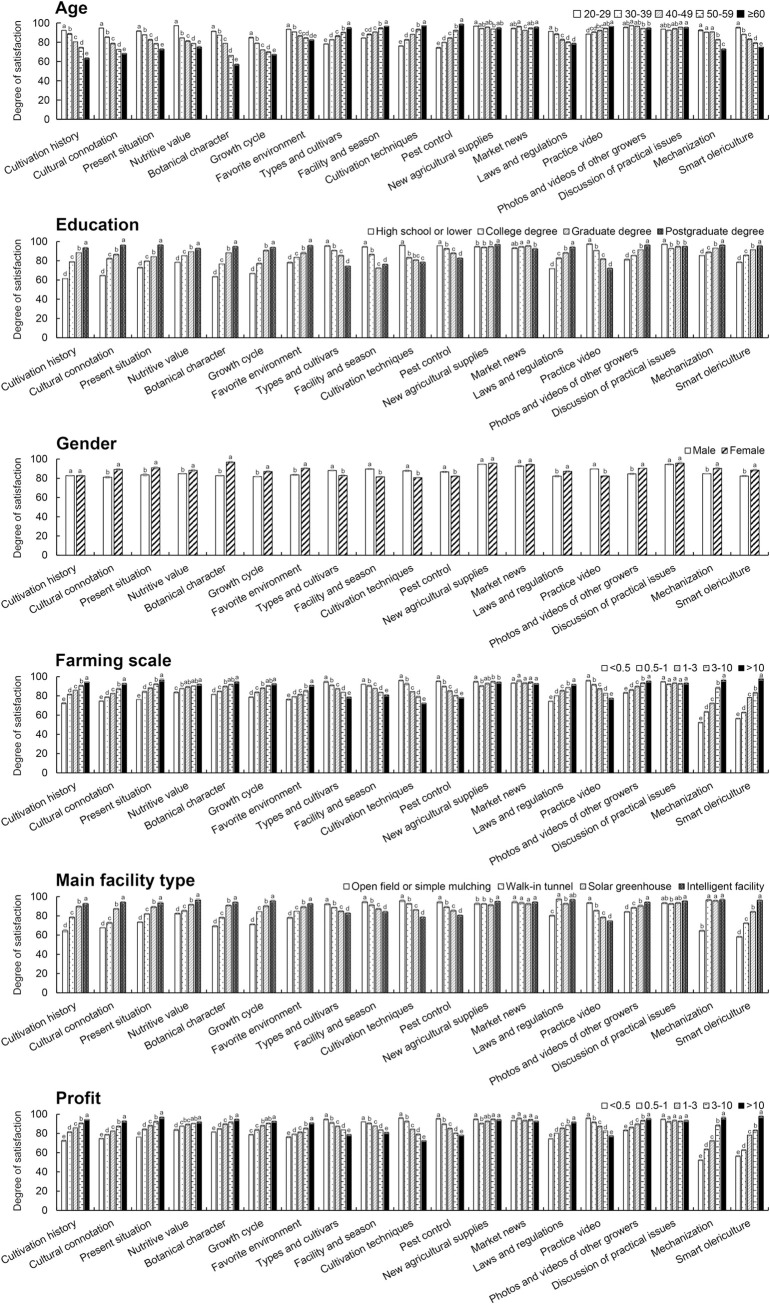
Importance attributed to different sectors of olericulture by vegetable farmers.

As shown in Fig 1, as the age of farmers and their level of education increase and as farming scale, facility advancement and profits decrease, “Cultivation history” and “Cultural connotations” of the cultural section; “The present situation” and “Nutritive value” of the social section; and “Botanical characteristics,” “Growth cycles” and “Favorable environments” of the theoretical section of the original MOOC and “Laws and regulations” of the social section and “Mechanization” and “Smart olericulture” of the comprehensive section of the modified MOOC are considered less important. On the other hand, “Types and cultivars,” “Facilities and seasons,” “Cultivation techniques” and “Pest control” are considered more important with the farmer age increases or education degree, the farming scale, facility advancement and profit decrease. “Photos and videos from other farms” of the practical section of the modified MOOC are considered more important with higher farmer education, greater farming scale, facility advancement and increasing profits, while the farmer’s age has no significant influence. Compared to male farmers, female farmers tend to make the same choices as farmers with a high level of education. Several subsections, including those focused on “New agricultural supplies” and “Market news” of the marketing section and “Discussion of practical issues” of the comprehensive section of the modified MOOC, were welcomed by all farmers.

### Effect of MOOC improvements on importance evaluations

**Fig 2:**
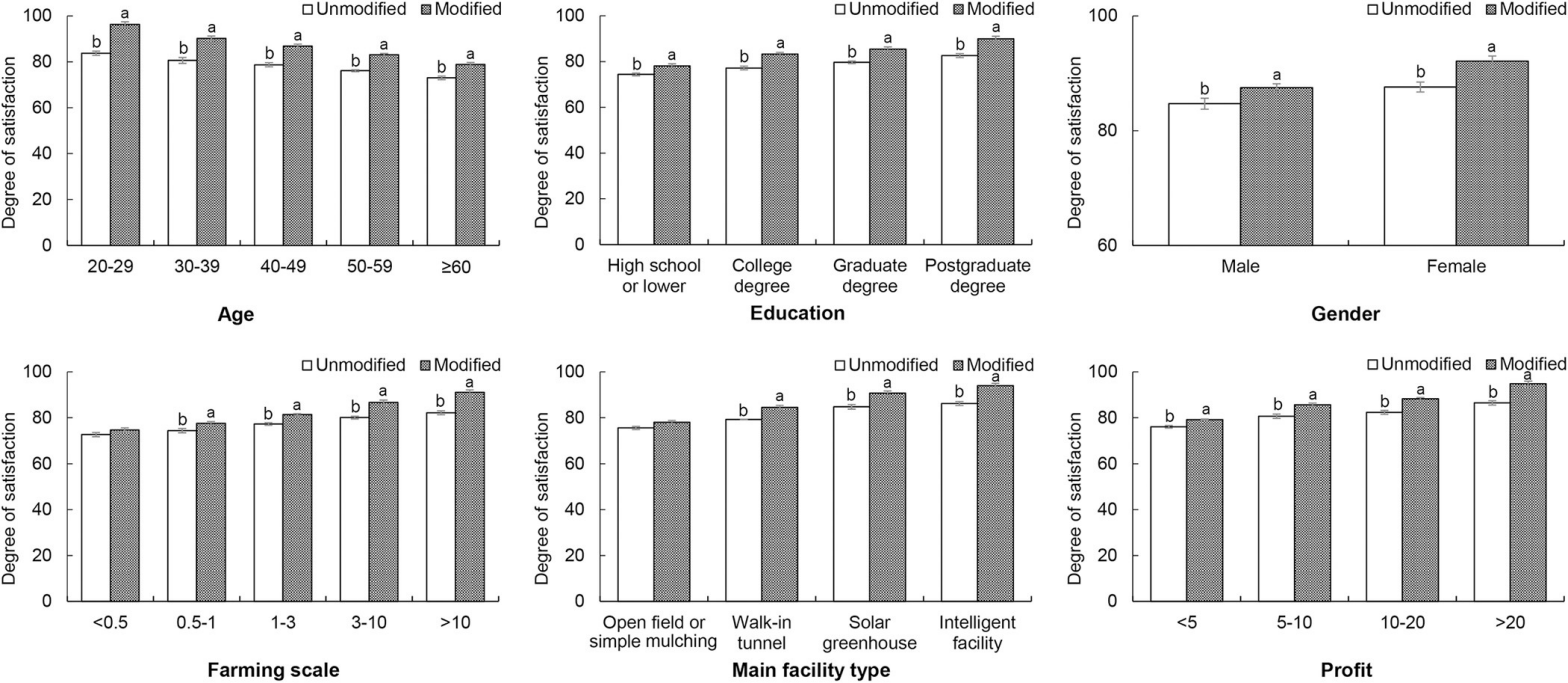
Satisfaction of different vegetable farmers.

The overall importance evaluations of the original and modified MOOCs from vegetable farmers with different backgrounds were also calculated (Fig 2). Generally, the addition of new sections to the MOOC significantly improved the importance evaluations of the surveyed farmers. The improvement was greater among younger farmers (12.6% of farmers 20–29 years of age and 5.9% of farmers ≥60 years of age), farmers with higher education degrees (7.4% of farmers with a postgraduate degree and 3.7% of farmers with a high school degree or lower), female farmers (4.5% of female farmers and 2.8% of male farmers), farmers with larger farms (8.9% of farmers with farming scales of >10 and 2.0% of farmers with farming scales of <0.5), farmers with more advanced facilities (7.8% of farmers with intelligent facilities and 2.8% of farmers adopting open field or simple mulching methods) and farmers earning higher profits (8.4% of farmers earning greater profits of >20 and 3.1% of farmers earning greater profits of <5).

### Effect of MOOC improvements on farmer profits

**Fig 3:**
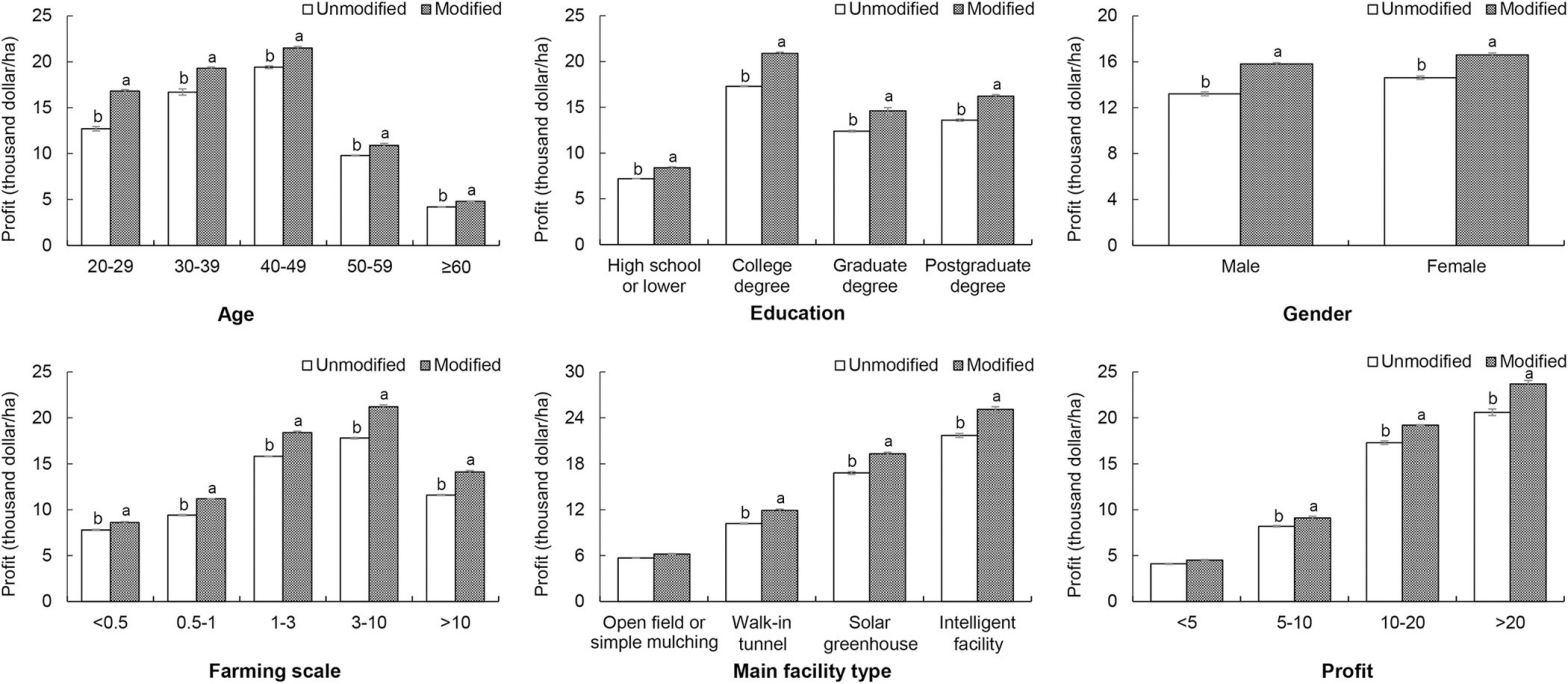
Profit increases before and after the MOOC among different vegetable farmers.

The effects of MOOC improvements on the profits of farmers with different backgrounds are shown in Fig 3. Similar to the effect on importance evaluations, the increase in profits was greater among younger farmers (4.1% of farmers 20–29 years of age and 0.6% of farmers ≥60 years of age), farmers with more advanced facilities (3.4% of farmers with intelligent facilities and 0.5% of farmers using open field or simple mulching methods) and farmers earning more profits (3.1% of farmers earning greater profits of >20 and 0.4% of farmers earning greater profits of <5); however, the profit-increasing effects of education, female gender and farming scales for the modified MOOC training differed from those obtained from the importance evaluation. The greatest increase in profit attributed to education was found for farmers with an undergraduate degree. The profit-increasing effect was greater for male farmers than female farmers. With an increase in farming scale, profits first increased and then decreased, and farmers with farming scales of 3–10 had the greatest profits.

## Discussion

In this study, 400 MOOC leaners were randomly selected from the MOOC learner groups and the survey was performed under a completely voluntary condition. In addition, Jiangsu Province has a large vegetable industry and has a long vegetable history for vegetable production. Thus, the results of the survey should be representative in other regions with similar conditions. However, it is worth pointing out that Jiangsu province is one of the most developed regions in China, the MOOC. Therefore, the promotion of agricultural MOOCs in other areas with poorer economic condition may be different and need further investigation.

The negative correlation between a farmer’s age and education level has been demonstrated by previous studies [21] and was also observed in this study. This should be a general rule in most developing countries. A negative correlation between a farmer’s age and the advancement main facility type was also observed, since there was a positive relationship between education and facility advancement. It should be noted that more advanced facilities require more complicated technology, requiring farmers to have a higher education degree. In many developing countries, women are generally subject to discrimination in agricultural areas and have an average less education than men [22, 23]. However, in our study, female farmers had higher education levels. On the one hand, the current enrollment rate of female students in colleges and universities is higher than that of male students in Jiangsu Province since the current college entrance examination system favors women [24]. On the other hand, female farmers need access to more technology to overcome their physical disadvantages. As a result, female farmers run larger farms and more advanced facilities and thus earn more profits. Furthermore, the positive correlations between facility advancement and farm scale and between facility advancement and profits indicate that advanced greenhouses are beneficial for improving economic returns and expanding farm scale.

In this study, we found that younger farmers and those with more education, larger farms, more advanced facilities and greater profits paid more attention to course sections associated with culture, society, and botanical theory and paid less attention to the practical sections of the MOOC. Theoretical sections help farmers understand reasons to use a certain method, and cultural and social sections provide information on how to improve the market value of vegetable products. In general, such sections can help farmers innovate during vegetable production. Yagüe-Perales et al. (2020) also found younger farmers and those with larger farms to be more innovative. In addition, the course section on “Laws and regulations” was considered more important among farmers with higher education levels, and such laws and regulations may be associated with environmental protection or food security [25]. Xin et al. (2022) found that gender, age and education level are key factors influencing farmers’ willingness to pay for environmentally friendly fertilizers [26]. Liu et al. (2021) also found farmer age to have a profound effect on farmers’ clean production behavior [21]. These results indicate that customized services should be provided when MOOCs are introduced to certain farmers.

As shown in Figs 2 and 3, the addition of new sections enhanced farmer learners’ interest in the MOOC overall and then improved the profits of these farmers. These effects were more remarkable for younger farmers and those with higher education levels, larger farms, more advanced facilities and greater profits. In sum, young, well-educated farmers operating larger farms should easily accept new knowledge and adopt new technologies [27, 28]. Thus, for this group of farmers, the improvement of training programs based on detailed surveys is more important than for other farmer groups.

Studies on the application of MOOCs on the farmer training are rare. Grunfeld and Ng (2013) suggested that the training effectiveness of open distance learning is similar to that of face-to-face training [29]. Patillo et al. (2021) demonstrated the efficacy of digital learning of southern Missouri farmers, and they also point out that the traditional extension approach such as one-on-one consulting or farm visits cannot be completely replaced by the online educational programming [30]. Yang and Yang (2022) found that farmer age, education level, and profits are influence factors of farmers’ interest of distance learning [12]. To our knowledge, our study is the first report to investigate how farmers’ demographic characteristics affect their interest in different sections of an agricultural MOOC. Our results provided meaningful information to improve agricultural MOOCs for farmer training. In the future, as mentioned by Corrales and Casas (2021), maps, photographs, sketches, diagrams and dynamic and attractive visual materials should be included to increase the attraction of agricultural MOOCs [31].

## Conclusions

### Theoretical implications

In the farmer population surveyed in this study, younger farmers had more education and ran more advanced facilities. Compared to male farmers, female farmers generally have more education, larger farms and greater profits. In addition, farmers with more advanced greenhouses usually manage larger farms and earn more profits. Among the different sections of the MOOC focused on olericulture, younger farmers and those with more education, larger farms, more advanced facilities and greater profits were more interested in cultural, social and theoretical knowledge and less interested in practical skills. This group of farmers was also interested in subsections focused on “Photos and videos from other farms”, “Mechanization” and “Smart olericulture”. Three subsections focused on “New agricultural supplies”, “Market news” and “Discussion of practical issues” were welcomed by all farmer learners.

### Practical implications

Based on an informal survey, eight new sections were added, and the modified MOOC was evaluated as more important and resulted in increased incomes for farmer learners. The improvement was more profound for younger farmers and those with more education, larger farms, more advanced facilities and greater profits. These results suggest that an individualized service should be provided and that careful surveys and improvements of current university MOOCs will be necessary, especially for highly educated farmers.

### Key lessons learnt

According to the Chinese traditional culture, farmers may not present their actual profits in the questionnaire. Therefore, other materials such as sale records should be included in the study.

### Limitations of this research

Jiangsu is one of the most developed provinces in China, so the result of this study may not represent the situation of the whole China and other developing countries. In the future, more agricultural MOOCs should be tested among a wider area.

## Supporting information

S1 Table The questionnaire. (DOCX)

S2 Table Values for the calculation of different levels and types of education, gender, and main facility types from the questionnaire. (DOCX)
