# Supplementary material for: University MOOC should be added with farmer interested sections and provide individualized service to adapt to farmer training
Source: PLoS One. 2023 Nov 2;18(11):e0288309. doi: 10.1371/journal.pone.0288309 (PMC10621842; doi:10.1371/journal.pone.0288309)
Supplement: S1 Table — (DOCX) [file pone.0288309.s001.docx]

**S1 Table The questionnaire**

| Age | 20-29□ 30-39□ 40-49□ 50-59□ ≥60□ | |
| --- | --- | --- |
| Education | high school or lower□ undergraduate degree□ graduate degree□  postgraduate degree□ | |
| Gender | Male□ Female□ | |
| Farming scale, ha | <0.5□ 0.5-1□ 1-3□ 3-10□ >10□ | |
| Main facility type | Open field or simple mulching (mulching film, low tunnel, shading)□ Walk-in tunnel□ Solar greenhouse□ Intelligent Facility (intelligent greenhouse and plant factory)□ | |
| Profit, Thousand dollars/ha | <5□ 5-10□ 10-20□ >20□ | |
| Cultual part | Cultivation history | Very important□ Important□ Somewhat important□  Not very important□ Not important at all□ |
|  | Cultural connotation | Very important□ Important□ Somewhat important□  Not very important□ Not important at all□ |
| Social part | Present situation | Very important□ Important□ Somewhat important□  Not very important□ Not important at all□ |
|  | Nutritive value | Very important□ Important□ Somewhat important□  Not very important□ Not important at all□ |
| Theoretical part | Botanical character | Very important□ Important□ Somewhat important□  Not very important□ Not important at all□ |
|  | Growth cycle | Very important□ Important□ Somewhat important□  Not very important□ Not important at all□ |
|  | Favorite environment | Very important□ Important□ Somewhat important□  Not very important□ Not important at all□ |
| Practical part | Type and cultivars | Very important□ Important□ Somewhat important□  Not very important□ Not important at all□ |
|  | Facility and season | Very important□ Important□ Somewhat important□  Not very important□ Not important at all□ |
|  | Cultivation techniques | Very important□ Important□ Somewhat important□  Not very important□ Not important at all□ |
|  | Pest control | Very important□ Important□ Somewhat important□  Not very important□ Not important at all□ |
| Marketable part | New agricultural supplies | Very important□ Important□ Somewhat important□  Not very important□ Not important at all□ |
|  | Market news | Very important□ Important□ Somewhat important□  Not very important□ Not important at all□ |
| Social part | Laws and regulations | Very important□ Important□ Somewhat important□  Not very important□ Not important at all□ |
| Practical part | Practice video | Very important□ Important□ Somewhat important□  Not very important□ Not important at all□ |
|  | Photos and videos of other growers | Very important□ Important□ Somewhat important□  Not very important□ Not important at all□ |
| Comprehensive part | Discussion of practical issues | Very important□ Important□ Somewhat important□  Not very important□ Not important at all□ |
|  | Mechanization | Very important□ Important□ Somewhat important□  Not very important□ Not important at all□ |
|  | Smart olericulture | Very important□ Important□ Somewhat important□  Not very important□ Not important at all□ |
