# Supplementary material for: University MOOC should be added with farmer interested sections and provide individualized service to adapt to farmer training
Source: PLoS One. 2023 Nov 2;18(11):e0288309. doi: 10.1371/journal.pone.0288309 (PMC10621842; doi:10.1371/journal.pone.0288309)
Supplement: S2 Table — (DOCX) [file pone.0288309.s002.docx]

**S2 Table Values for the calculation of different levels and types of education, gender, and main facility types from the questionnaire**

|  | **Different types** | **Assigned value** |
| --- | --- | --- |
| Education | high school or lower  undergraduate degree  graduate degree  postgraduate degree | 25  50  75  100 |
| Sex | Male  Female | 0  1 |
| Main facility type | Open field or simple mulching (mulching film, low tunnel, shading)  Walk-in tunnel  Solar greenhouse  Intelligent Facility (intelligent greenhouse and plant factory) | 25  50  75  100 |
